# Constrained stochastic optimal control with learned importance sampling: A path integral approach

**DOI:** 10.1177/02783649211047890

**Published:** 2021-10-12

**Authors:** Jan Carius, René Ranftl, Farbod Farshidian, Marco Hutter

**Affiliations:** 1 Robotic Systems Lab, ETH Zurich, Switzerland; 2 Intel Labs, Munich, Germany

**Keywords:** Stochastic optimal control, sampling-based planning, importance sampling

## Abstract

Modern robotic systems are expected to operate robustly in partially unknown environments. This article proposes an algorithm capable of controlling a wide range of high-dimensional robotic systems in such challenging scenarios. Our method is based on the path integral formulation of stochastic optimal control, which we extend with constraint-handling capabilities. Under our control law, the optimal input is inferred from a set of stochastic rollouts of the system dynamics. These rollouts are simulated by a physics engine, placing minimal restrictions on the types of systems and environments that can be modeled. Although sampling-based algorithms are typically not suitable for online control, we demonstrate in this work how importance sampling and constraints can be used to effectively curb the sampling complexity and enable real-time control applications. Furthermore, the path integral framework provides a natural way of incorporating existing control architectures as ancillary controllers for shaping the sampling distribution. Our results reveal that even in cases where the ancillary controller would fail, our stochastic control algorithm provides an additional safety and robustness layer. Moreover, in the absence of an existing ancillary controller, our method can be used to train a parametrized importance sampling policy using data from the stochastic rollouts. The algorithm may thereby bootstrap itself by learning an importance sampling policy offline and then refining it to unseen environments during online control. We validate our results on three robotic systems, including hardware experiments on a quadrupedal robot.

## 1. Introduction

We see today an increasing proliferation of mobile robotic systems in real-world scenarios. To be able to deploy autonomous machines in our vicinity, there is a clear need for robust control designs that can handle unforeseen situations and perform safely in complex environments. In this work, we introduce a sampling-based control algorithm for a general class of stochastic dynamical systems. Our method highlights a path to fulfill the requirements on modern control designs using a combination of reactive online control and data-driven offline learning.

It is usually intractable to analytically devise a globally stable feedback controller for a system with multiple, non-linearly coupled states and inputs, that is operating in a partially unknown environment. Accordingly, in recent years, almost all sophisticated control designs for complex robotic systems in real-world scenarios can be associated with one of two fundamental design strategies: model-based optimal control (OC) and data-driven reinforcement learning (RL). Both strategies come with their own set of difficulties and compromises.

OC methods require the specification of an underlying model, where the complexity of the model must strike a balance between multiple, conflicting objectives (Chung et al., 2016): On the one hand, the model must capture the essential dynamic behavior of the system and its interaction with the environment. On the other hand, it needs to be simple enough to create a smooth optimization landscape such that the solution is not sensitive to the initialization and gradient updates do not become trapped in an undesired local minimum. The dependence on gradients limits the types of scenarios that can be modeled and requires that derivatives can be computed. The model also needs to be computationally lean to achieve the fast update rates of the controller that are required to achieve robustness against disturbances. In contrast, data-driven methods, such as RL, in general do not require the manual specification of a model, but require careful attention to the data generation process to find a balance between sample complexity and sufficient realism. This is especially true when applying RL to real robotic systems (Peters et al., 2016). How to systematically devise a suitable data generation pipeline for learning complex control policies is an open problem and an active field of research.

Furthermore, data-driven methods have only become viable candidates for online control of higher-dimensional systems after introducing function approximation, i.e., fitting a parametrized control law, which may be evaluated rapidly online, to the data generated in a costly offline process (Bertsekas, 2005; Sutton and Barto, 1998). The decisive advantage of learning a controller from data is that there are minimal restrictions to modeling the robot and its environment because the model does not need to be in an analytical form. Hence, a general-purpose physical simulator is often used in the robotics context. Subsequently, it requires little additional effort to represent complex scenarios such as uneven ground geometries or random disturbances in the problem formulation and prepare the learned policy for these contingencies.

Beyond modeling of the system and environment, a mathematical representation for the task at hand must be formulated when constructing an OC or RL problem. In data-driven algorithms, the task specification is almost exclusively embedded in the cost (or reward) function. For OC the complexity of the cost function is usually limited due to the differentiability requirement and the danger of local minima. However, a powerful feature of OC is its ability to respect constraints. Such conditions can be used to encode a goal, safety boundaries, or to prioritize a hierarchical set of tasks, all of which is typically difficult to guarantee in an RL algorithm.

In this article, we introduce a way to combine the ease of modeling realistic scenarios with the constraint-handling abilities and reactiveness of online OC. Our work is founded on the theory of path integral stochastic optimal control (PISOC) (Kappen, 2005; Todorov, 2006): At its core, our method infers the optimal input by drawing trajectory samples from a stochastic system. The underlying dynamics model may thereby be represented by an arbitrarily complex formulation, such as a complete simulator. These noisy rollouts provide a principled way to encode uncertainty, which translates into robustness of the optimized control commands against disturbances, model mismatches, and imperfect actuation. The stochastic model also ensures exploration into unseen states, thereby providing information on favorable locations in the cost landscape without the need for a smooth and differentiable problem description.

While sampling in continuous, high-dimensional spaces is generally prone to failure, we use two mechanisms in our work that reduce the sampling complexity and allow us to execute our algorithm in real-time. First, we extend the path integral (PI) formalism to explicitly handle equality constraints. Our results show that such constraints can be dealt with using a projection approach, which significantly reduces the sampling space and provides a powerful mechanism to introduce domain-specific knowledge. Second, we use importance sampling by integrating a known (sub-optimal) controller into the sampling procedure. This underlying policy steers the sampling distribution and increases the informational content for each sample. If such an ancillary controller is not known or not easily obtainable, e.g., by solving a simpler deterministic OC problem, it is possible to use the exact same PISOC algorithm in an offline setting to train a feedback policy, which eventually becomes a suitable importance sampler.

Compared with deterministic model predictive control (MPC), our algorithm benefits from advantages that are typically attributed to data-driven methods: as no derivatives of the system dynamics are required, we achieve a high degree of flexibility in modeling and can directly integrate complex physics engines into the controller. This allows us to model disturbances beyond uncertain dynamics, for example, arbitrary obstacles and uneven ground geometry. Furthermore, the stochastic problem setting creates an automatic balance between robustness and performance, which would otherwise require careful tuning of the cost function. It also leads to exploration, which is difficult to achieve in deterministic control, particularly if exploration needs to happen across non-smooth events such as contact switches. Finally, the sampling procedure, which consumes the bulk of the computation time, can be parallelized entirely on modern hardware.

We reap these benefits while being able to retain several merits from model-based OC methods. Most importantly, we can respect equality constraints exactly through a projection approach while allowing the flexible integration of other constraints in the dynamics or cost function. Our algorithm is fast enough to be run online and thus lends itself naturally to receding horizon control. Combined with its ability to include any existing control law as an ancillary controller, we can flexibly build on established control schemes and advance them with robust online adaptation through our method. Such a combination can be powerful in the context of robots interacting with their perceived environment, for example, by fusing an existing model-based proprioceptive controller with our sampling procedure to achieve online adaptation to terrain variations and obstacles. In addition, if an ancillary controller is too slow for online execution or even no such feedback policy is known *a priori*, the same sampling procedure can be used in an imitation learning (IL) or RL setting to bootstrap itself. Therefore, our algorithm can be viewed as an online sampling-based planner or an offline learning algorithm depending on the execution setting.

Our developed method is broadly applicable to a wide range of robotic systems. In this work, we highlight its performance on three exemplary systems of increasing difficulty level: a fixed-base robotic arm, a mobile ball-balancing robot, and a quadrupedal walking robot. In particular, legged robots belong to the most challenging class of robotic systems owing to their strongly non-linear and high-dimensional dynamics and non-smooth contact switching behavior. Moreover, they are intended to be operated in the most challenging and uncertain environments. For this reason, we choose this class of robots as one of the three subjects of our experimental study. Our experiments indicate that our method’s advantages in terms of flexible importance sampling and ability to handle constraints become especially pronounced for walking systems.

### 1.1 Statement of contributions

This article makes a case for solving stochastic OC problems using a hybrid approach based on classical control theory, receding horizon PI control, and machine learning. We provide a way to reap the modeling flexibilities of sampling-based methods while retaining the constraint-handling and real-time capabilities of model-based MPC. In that respect, the PI formality provides a natural way to combine policy learning ideas with classical model-based or hand-tuned controllers. The resulting algorithm presented in this article can be applied to the general class of non-linear, control-affine dynamical systems. The computed solution respects state-input equality constraints.

Specifically, this article presents the following contributions.

- We extend the PI algorithm to handle time-variant state-input equality constraints.
- We demonstrate that the PI sampling complexity for unstable systems is reduced by introducing constraints and importance sampling.
- We validate that our measures yield a sampling efficiency that is high enough for online control of a quadrupedal robot.
- We showcase how the developed algorithm can also be used in an offline setting to (pre-)train a sampling policy.
- We illustrate how our PISOC algorithm can exploit the freedom in modeling by using a physics simulator to provide state trajectories in complex environments.
- We conduct experiments on a quadrupedal robot in combination with a stepping stone terrain to demonstrate the effectiveness of our real-time sampling algorithm.

## 2. Related work

The core of our algorithm is based on PISOC. The theory of PI control (Kappen, 2005; Theodorou, 2015; Todorov, 2006) suggests a mathematically elegant procedure to determine optimal controls while avoiding parametrized representations of the value function and control policy. Under a few regularity assumptions and the application of the Feynman–Kac formula (see Øksendal, 2003), the theory states that the evaluation of the OC at any given state is given by a conditional expectation value.

In discrete state and action spaces, it is conceivable that the expectation can realistically be evaluated for the finite set of state–action pairs (Azar et al., 2012). However, for most stochastic control problems computing this expectation in closed form is intractable. To address this problem, different analytical options have been proposed in the literature, for example, the Laplace approximation (Kappen, 2005) or the method of implicit sampling (Morzfeld, 2015). These methods presume that an analytical description of the underlying system dynamics is available. If this is not the case, the most frequently used approach for estimating the expectation value is Monte Carlo sampling. Drawing a sample in this context means computing a rollout of the stochastic system dynamics over the specified time horizon. Except for systems with limited complexity, the corresponding sampling space is vast, thus rendering nave sampling schemes unusable owing to exceedingly high variance of the estimation.

Intuitively, what makes sampling difficult is that we are hoping to discover a sensible behavior from a system that is purely driven by noise. The application of Girsanov’s change-of-measure formula provides a remedy by revealing how the sought expectation value can also be obtained from sampling a stochastic process that is driven by noisy controls from an arbitrary controller (Doucet et al., 2001). Such a controller can be the solution from the previous iteration of the algorithm or also any externally given (feedback) policy. Variations of this idea have been explored in the literature, for example, Stulp and Sigaud (2012), Thijssen and Kappen (2015), Kappen and Ruiz (2016), and Williams et al. (2017, 2018b). In our work, we also make extensive use of this form of importance sampling.

Another family of methods that avoid sampling in high-dimensional action spaces are those related to the seminal Policy Improvement with Path Integrals (PI^2^) method by Theodorou et al. (2010). Their key idea is to reformulate the stochastic optimal control (SOC) problem in a way such that the control action becomes a set of parameters of a feedback controller. The cardinality of the sampling space thereby reduces to the size of a single parameter vector, which is typically much smaller than that of the original control space (input dimension times number of time steps). Many works have followed up on the idea of optimizing parameters through PI ideas, for example, Buchli et al. (2011), Kalakrishnan et al. (2011), and Pastor et al. (2011).

In a second line of work, an information-theoretic viewpoint on SOC was developed in the literature, which avoids the notion of a PI. From this perspective, SOC can equivalently be formulated as an inference problem (Kappen et al., 2012; Theodorou and Todorov, 2012; Todorov, 2009; Toussaint, 2009). Therein, the optimal policy is computed through the minimization of the Kullback–Leibler (KL) divergence between the optimal state distribution and the state distribution that is arising from applying the current policy. Although still not analytically solvable, this minimization can be attempted iteratively, yielding practical algorithms for optimizing parametrized feedback controllers. In our work, we build on ideas from Gómez et al. (2014), Thijssen and Kappen (2015), and Kappen and Ruiz (2016) to devise such a built-in optimization mechanism for learning a parametrized feedback policy that is subsequently employed as an importance sampling mechanism.

The cross-entropy view of PI naturally suggests a probability-weighted update rule to the learnable parameters of a sampling policy. The idea of using probability-weighted averaging rather than gradient estimation is shared with numerous approaches that derive from covariance matrix adaptation evolution strategy (CMA-ES) and similar cross-entropy arguments (Stulp and Sigaud, 2012). More interrelations between SOC, online learning, and statistical inference are investigated by Rawlik et al. (2012) and Wagener et al. (2019). Interestingly, the learning component of our method also bears similarity to the popular soft actor–critic (SAC) (Haarnoja et al., 2018), an off-policy RL algorithm. A difference is, however, that we use an online estimate of the value function based on the current set of samples instead of learning a Q -function. Furthermore, we do not employ function approximation except for the learned policy itself. There are many instances of PI control in a robotic context, both for parameter estimation as well as direct control, for example Sugimoto and Morimoto (2011), Rombokas et al. (2012), Theodorou et al. (2010), Rawlik et al. (2013), Williams et al. (2017), Heijmink et al. (2017), Okada and Taniguchi (2018), Hoeller et al. (2019), and Abraham et al. (2020). In typical use cases, the modeled optimization problem is an unconstrained one. Many successful PI control applications have been shown on systems with a purposefully designed input space or where a set of default inputs already yields stable behavior (Goldfain et al., 2019; Theodorou et al., 2010). The use of iterative learning and importance sampling allows us to directly apply our method to the system’s continuous input space without an intermediate representation, such as movement primitives.

One disadvantage of using a sampling-based over a gradient-based OC algorithm is an increase in computation cost and, accordingly, a slower algorithm. Fortunately, the nature of the PI algorithm lends itself naturally to parallel computation because the bulk of the computation time is spent in forward-simulating system trajectories under independent noise realizations. Therefore, parallelization of the sampling procedure is the primary mechanism to achieve the required computational efficiency for running PISOC solvers in real-time. Williams et al. (2017) have proposed an efficient GPU implementation for online PI control, termed model predictive path integral (MPPI). Although our work does not claim to be of the same implementation maturity, we follow the same general idea of using receding horizon PI control with parallel computation to offset the computational burden of the sampling-based approach.

## 3. Preliminaries: PI control

We begin by recalling the theoretical background of our method and the notation used throughout the article. The underlying theory of PI control resides on linearly solvable Markov decision processes and has been introduced by Kappen (2005) and Todorov (2006).

### 3.1. Problem formulation

PI methods operate on SOC problems defined by the control-affine dynamics

(1) dx=f(t,x)dt+G(t,x)(udt+Q(t,x)dw)

which are driven by exogenous input vector $u\in R^{n_{u}}$ and normalized white noise w of the same dimension. The flow map f defines the dynamics of the system’s state $x\in R^{n_{x}}$ absent of any inputs and stochasticity. The system’s evolution (1) is a Brownian motion with diffusion term GQ and drift f+Gu . This formulation ensures that the noise enters through the input channel only, which is a requirement of the PI formalism and means that only controllable degrees of freedom will be perturbed .

The scalar cost function C of our problem takes the form of an expectation of path costs S over all possible noise realizations

(2) $$C(t_{0},x_{0},u(t_{0}..t_{f}))=E_{x_{0}}\{S(t_{0},x(\cdot ),u(\cdot ))\}$$

(3) $$S(t_{0},x(\cdot ),u(\cdot ))=\Phi (x_{t_{f}})+\int_{t_{0}}^{t_{f}}(V(x)+\frac{1}{2}u^{⊤}\mathrm{Ru})d\tau$$

where $E_{x_{0}}$ denotes the expectation over all trajectories that start at $x_{0}$ and are governed by the dynamics (1). We use the notation $t_{0}\ldots t_{f}$ to denote the range of times from $t_{0}$ to $t_{f}$ . We assume a positive-definite and symmetric control cost matrix R . The potential V(x) and final cost Φ(x) may be arbitrary function s of the state and can include areas with infinite potential, for example to model obstacles or unsafe regions.

In short, the SOC problem can be stated as

(4) $$\begin{array}{cc} \underset{u(t_{0}\ldots t_{f})}{\mathrm{minimize}}\; & C(t_{0},x_{0},u(t_{0}\ldots t_{f}))\\ \mathrm{subject}\;\mathrm{to} & x(t_{0})=x_{0}\\ & \mathrm{Dynamics}(1) \end{array}$$

### 3.2. Optimal cost-to-go

The optimal cost-to-go J is a function that represents the minimal cost (in expectation) that can be achieved when starting from state x at time $t\in [t_{0},t_{f}]$ :

(5) $$J(t,x)=\underset{\begin{array}{c} u(t\ldots t_{f}) \end{array}}{\min}E_{x(t)}\left\{S(t,x(\cdot ),u(\cdot ))\right\}$$

Solving the OC problem entails finding the optimal cost-to-go. The optimality conditions of this minimization lead to a non-linear partial differential equation (PDE) named Hamilton-Jacobi-Bellman (HJB) equation. Finding a global solution to the HJB is generally intractable and not expedient for control applications. Luckily, under the condition that the noise magnitude is inversely related to the control cost,

(6) $$QQ^{⊤}=\gamma_{J}R^{-1}$$

i.e., the control effort is cheap in directions where the noise is strong, $\gamma_{J}>0$ being a proportionality constant, the second-order terms in the HJB cancel. Under these conditions, there exists a variable transformation of the form

(7) $$J(t,x)=-\gamma_{J}\log\psi (t,x)$$

that linearizes the PDE in terms of ψ . The exponentiated cost-to-go function ψ is commonly known as the desirability function.

The second practical aspect that eases the computational demands is that control applications typically only require the OC at the current state. In combination with the mentioned linearization of the HJB, it can be shown (Kappen, 2005) that the point-wise optimal cost-to-go can be written as an expectation over all possible paths that the uncontrolled (passive control policy $\pi_{p}(t)=0\;\forall t$ ) stochastic dynamics may take

(8) $$J(t,x)=-\gamma_{J}\log E_{\pi_{p}}\left\{\exp\left(-\frac{1}{\gamma_{J}}S(t,x(\cdot ),u(\cdot ))\right)\right\}$$

The operator $E_{\pi }$ thereby signifies the expectation over all trajectories that start at $x_{0}$ and are governed by policy π and dynamics (1).

The optimal input $u^{*}$ at the current time and state is related to the derivative of the optimal cost-to-go via the relationship

(9) $$u^{*}=-R^{-1}G^{⊤}(\partial_{x}J{)}^{⊤}$$

This formula is generally true for continuous-time OC problems with quadratic control cost and control-affine dynamics (Bertsekas, 2005). Thijssen and Kappen (2015) prove that in our stochastic setting the derivative of J may also be written as an expectation operator, which yields a formula for the OC analogous to the optimal cost-to-go:

(10) $$\begin{array}{c} u^{*}(t,x)=\frac{1}{\psi (x,t)}\underset{t\prime \ldots t}{\lim}E_{\pi_{p}}\{\int_{t}^{t\prime }\frac{1}{t\prime -t}dw\\ \cdot \exp\left(-\frac{1}{\gamma_{J}}S(t,x(\cdot ),u(\cdot ))\right)\} \end{array}$$

## 4. Method

Having established the theoretical foundations, we now present our core method (Section 4.1) and several extensions that make PI control applicable to a wide range of robotic systems. Of these extensions our results show that temperature tuning (Section 4.2) and importance sampling (Section 4.3) are crucial, whereas the learning aspect (Section 4.4) is a useful but optional enhancement.

### 4.1. Constrained PI control

The first crucial ingredient of our method is the generalization of the PI formalism to constrained problem settings. Williams et al. (2018a) mentioned that inequality constraints that are purely a function of the state, such as joint limits, can be handled through suitable barrier or indicator functions inside the potential V(x) . L imits on the control commands may be enforced by quadratic penalties to keep the overall path cost quadratic in u .

In the following, we focus specifically on the treatment of state-input equality constraints because these conditions can be handled through a projection scheme. This is particularly beneficial for our method because it results in a dimensionality reduction of the sampling space. Detailed derivations for this section can be found in Appendix A.

We consider problem (4) with a slightly more complex path cost

(11) $$\begin{array}{c} S(t_{0},x(\cdot ),u(\cdot ))=\Phi (x_{t_{f}})\\ \;+\int_{t_{0}}^{t_{f}}(V(x)+r^{⊤}(x)u+\frac{1}{2}u^{⊤}\mathrm{Ru})d\tau \end{array}$$

Similar to V , the additional coupling term r(x) can be an arbitrary function of the state. Furthermore, our problem includes an additional ( $n_{c}$ -dimensional) control-affine equality constraint of the form

(12) c(t,x)+D(t,x)u=0

under the assumption that $D\in R^{n_{c}\times n_{u}}$ has full row-rank for all attainable states and $c\in R^{n_{c}}$ being an arbitrary function . To keep our notation compact, we henceforth drop the explicit t and x dependence for most terms.

The resulting definition of the cost-to-go is analogous to (5), except that the minimization is constrained by the additional condition (12), which leads to the expression

(13) −(∂tJ)=minu(t)s.t.(12)(12u⊤Ru+r⊤(x)u+(∂xJ)(Gu))+V(x)+(∂xJ)f+12Tr((∂xxJ)GQQ⊤G⊤)

We can now use the method of Lagrange multipliers and rules from Itô calculus to derive optimality conditions that respect the dynamics (1) and the constraint (12). For the input this procedure results in the expression

(14) $$u^{*}=\pi_{c}(x)-(I-\tilde{D})R^{-1}G^{⊤}(\partial_{x}J{)}^{⊤}$$

whereas the constrained HJB equation reads

(15) $$\begin{array}{c} -(\partial_{t}J)=\frac{1}{2}\pi_{c}^{⊤}R\pi_{c}+r^{⊤}\pi_{c}+V(x)+(\partial_{x}J)(G\pi_{c}+f)\\ +(\partial_{x}J)G\left(\frac{-1}{2}(I-\tilde{D})R^{-1}\right)G^{⊤}(\partial_{x}J{)}^{⊤}\\ +\frac{1}{2}\mathrm{Tr}((\partial_{\mathrm{xx}}J)\mathrm{GQ}Q^{⊤}G^{⊤}) \end{array}$$

Here, we have used the following projection operators and shorthand symbols to keep the notation concise:

(16) $$D^{†}:=R^{-1}D^{⊤}(DR^{-1}D^{⊤}{)}^{-1}$$

(17) $$\tilde{D}:=D^{†}D$$

(18) $$\pi_{c}(x):=-(I-\tilde{D})R^{-1}r-D^{†}c$$

When comparing (14) with (9), one can observe that the control is constraint-satisfactory by design due to the projection operator $\left(I-\tilde{D}\right)$ . Note that we denote open-loop controls by u and distinguish them from feedback policies π that generally depend on the state. Here $\pi_{c}$ is the constrained equivalent to the passive $\pi_{p}$ policy above, i.e., it minimizes the control cost subject to the constraints but is ignorant of the state cost .

As indicated in the preliminaries, there exists a log transformation $J(t,x)=-\gamma_{J}\log\psi (t,x)$ for the optimal cost-to-go, which defines the desirability function ψ . This transformation, together with a structural assumption about the noise characteristics

(19) $$\gamma_{J}(I-\tilde{D})R^{-1}=QQ^{⊤}$$

linearizes the HJB in terms of ψ . This linearization procedure gives rise to the name “linearly-solvable Markov decision problems” (Todorov, 2006). The standard assumption taken in (19) means that the noise magnitude is inversely proportional to the control cost and that noise cannot act in constrained directions. The parameter $\gamma_{J}>0$ relates the overall noise magnitude to the control cost. As noise is usually regarded as a driver of exploration rather than a property of the underlying system, (19) merely dictates how the noise covariance Q should be calculated.

According to the Feynman–Kac formula, the point-wise solution of this linear PDE can be estimated by an expectation operator (Kappen, 2005)

(20) $$\psi (t,x_{0})=E_{\pi_{c}}\left\{\exp\left(-\frac{1}{\gamma_{u}}S(t,x(\cdot ),u(\cdot ))\right)\right\}$$

with scalar $\gamma_{u}>0$ where the expectation is over all trajectories that start at x at time t and are governed by the diffusion

(21) $$dx=(f+G\pi_{c})dt+\mathrm{GQ}dw$$

The expectation (20) may be computed by any desired statistical method. As our algorithm applies to general system dynamics f,G whose functional form may not be known, we choose to evaluate the expectation through Monte Carlo sampling. Importantly, this choice imposes minimal restrictions on the dynamics and cost terms and only requires that we have access to the evaluations of these quantities. Notably, this permits the use of implicitly given system dynamics, for example, through the evaluation of a physics simulator.

Finally, the optimal input to apply to the system at the current state follows from the state derivative of the value function and reads

(22) $$\begin{array}{c} u^{*}(t,x)=\frac{1}{\psi (x,t)}\underset{t\prime \to t}{\lim}E_{\pi_{c}}\{\int_{t}^{t\prime }\frac{1}{t\prime -t}Qdw\\ \cdot \exp\left(-\frac{1}{\gamma_{u}}(\Phi (x_{t_{f}}\right)\\ +\int_{t}^{t_{f}}\left(\frac{1}{2}\pi_{c}^{⊤}R\pi_{c}+r^{⊤}\pi_{c}+V(x)\right)d\tau ))\} \end{array}$$

where the first term in the expectation is the noisy input applied to the system in the *first* instance of the respective sampled path. Note that the computation of the OC is numerically robust against diverged samples because their high cost automatically makes their contribution to the expectation negligible. Alternatively, and with the same effect, failed samples may directly be discarded. The exact definition of failed samples is dependent on the problem context but generally corresponds to reaching a state from which the system cannot recover.

### 4.2. Automatic temperature tuning

When computing the expectation by Monte Carlo Sampling of random trajectories, the formulas (20) and (22) can be interpreted as a soft-max operation on the negative trajectory cost with $\gamma_{u}$ as the temperature parameter. This temperature determines how sharply the algorithm discriminates between high- and low-cost samples.

Strict adherence to the PI theory requires setting $\gamma_{u}=\gamma_{J}$ , i.e., the soft-max temperature is directly related to the noise level in the system. However, experience shows that, for most scenarios, such a direct implementation is a poor choice because the magnitude of the cost terms R,V can be arbitrary (Williams et al., 2018a). Bad scaling may lead to numerical problems because the evaluation of the exponential can encounter numerical under- or overflow.

We use two methods to reduce the dependency on problem-specific scales. First, the weighted contribution $w_{i}$ of each sample i is normalized by the minimum and maximum costs of the entire batch of drawn samples within the exponential:

(23) $$w_{i}=\exp\left(-\frac{1}{\gamma_{u}}\frac{S_{i}-\underset{j}{\min}[S_{j}]}{\underset{j}{\max}[S_{j}]-\underset{j}{\min}[S_{j}]}\right)$$

The computation of the optimal input, corresponding to (22), then reads

(24) $$u^{*}=\sum_{i}(\epsilon_{i}w_{i})/\sum_{i}w_{i}$$

where $\epsilon_{i}$ is the initial noise increment applied to sample i and the sum runs over all samples $n_{\mathrm{total}}$ .

The second mechanism we introduce is an automatic adjustment of the temperature coefficient $\gamma_{u}$ . We initialize $\gamma_{u}=\gamma_{J}$ according to the theory, but then allow it to change depending on the effective sample size

(25) $$n_{\mathrm{eff}}=\sum_{i}w_{i}/n_{\mathrm{total}}$$

This measure indicates the fraction of samples that are effectively contributing to the final OC. We find that $n_{\mathrm{eff}}=50\%$ is a reasonable target value that strikes a good compromise between choosing very few samples (high variance solution) and allowing many inferior samples to contribute to the optimal input. Our simple update strategy

(26) $$\gamma_{u}\leftarrow \left\{ \begin{array}{cc} 0.9\gamma_{u} & \mathrm{if}\;n_{\mathrm{eff}}<0.5\\ 1.1\gamma_{u} & \mathrm{if}\;n_{\mathrm{eff}}\geq 0.5 \end{array}\right.$$

automatically adapts $\gamma_{u}$ each iteration to the current cost scale without introducing additional problem-specific tuning parameters.

### 4.3. Importance sampling

In an online control application of our algorithm, it is critical that updates to the OC are computed as quickly as possible. Most of the computation time is spent in the sampling procedure for evaluating the expectation (22).

As with other Monte Carlo methods, the required number of samples for a reliable statistical estimate depends on the distribution from which the samples are drawn. The mechanism for shaping the sampling distribution is called importance sampling. The following sections outline two techniques that we use in our controller to improve the efficiency of the sampling procedure.

#### 4.3.1. Importance sampling with an ancillary controller

When estimating the expectation (22) with Monte Carlo samples, the trajectory distribution induced by the stochastic dynamics and the default^1^ policy $\pi_{c}$ is likely inefficient because an overwhelming majority of samples will end up in high-cost areas of the state space that are irrelevant to the optimal solution. When only a few “lucky” samples dominate, the Monte Carlo estimator has an extremely high variance in estimating the OCs. The negative effect on robot control is two-fold in this case. First, the optimal input trajectory computed by an iteration of the algorithm is very noisy due to the independent noise terms Qdw in each time step, which are only averaged over a small number of samples. Second, the solution in the next iteration may be significantly different, leading to a jump in the reference state and input that is passed to the robot.

Importance sampling with an ancillary controller can be used to change the sampling distribution to provide an estimator of lower variance. An active importance sampling policy, $\pi_{a}$ , that still satisfies the problem’s constraints is generally of the form

(27) $$\pi_{a}=(I-\tilde{D}){\tilde{\pi }}_{a}-D^{†}c$$

By construction the resulting controls will always remain constraint satisfactory. The likelihood ratio (or Radon–Nikodym derivative in this case) that corrects for the shifted sample distribution can be computed through Girsanov’s theorem (Theodorou and Todorov, 2012). As a result, we may sample from the controlled dynamics

(28) $$dx=(f+G\pi_{a})dt+\mathrm{GQ}dw$$

and compute the desirability function as

(29) $$\begin{array}{c} \Psi (t,x)=E_{\pi_{a}}\{\exp(\frac{-1}{\gamma_{u}}(\int_{0}^{t}\left(\frac{1}{2}\pi_{a}^{⊤}R\pi_{a}+r^{⊤}\pi_{a}+V\right)dt\\ +\Phi +\int_{0}^{t}(\pi_{a}-\pi_{c}{)}^{⊤}\mathrm{RQ}dw))\} \end{array}$$

If $\pi_{a}$ is stabilizing the system to remain in low-cost regions of the state space, the additionally arising control cost terms will be more than compensated for by a much lower state cost. Therefore, the introduction of the importance sampling policy $\pi_{a}$ results in much more informative samples.

Conveniently, the controller ${\tilde{\pi }}_{c}$ can take arbitrary forms. Suitable candidates that we use in this work are, for example: (a) last iteration’s optimal input sequence, (b) a model-based fallback controller, and (c) a learned sampling policy.

#### 4.3.2. Elite samples

Another convenient trick that reduces the computational burden of simulating stochastic trajectories is the concept of elite samples, which is inspired by particle filtering and evolutionary algorithms (De Jong, 1975).

In a receding horizon application of the SOC method, the starting states of consecutive calls to our controller are usually very close to each other. Therefore, it is likely that the input sequence of successful samples in the previous iteration is likely to produce good results in the current iteration too.

What we do, therefore, is to carry over the input sequence of the best samples from the previous iteration and re-evaluate their associated path costs. We find that saving approximately the top 10% of samples for the next iteration already results in more consistent expectation values and significantly smoother control commands across iterations.

### 4.4. Learning an importance sampling policy

So far, the introduced importance sampling techniques have assumed that a reasonable control sequence is available, either from the previous iteration of the algorithm or from an external ancillary controller.

In theory, even when starting without a known control sequence or policy, the repeated application of the PI algorithm should yield successively better control sequences and, subsequently, more informative samples each iteration. However, this improvement cycle can fail to start if the majority of rollouts fail to find an appropriate solution initially. The problem is significantly exacerbated in the case of unstable systems where the chances of finding stabilizing controls are diminishing and where previously stable (open-loop) control sequences may crash when subjected to a different noise realization.

A remedy to these issues is feedback control: a state-dependent controller $\pi_{a}(t,x)$ can be used during the stochastic rollouts to reject disturbances instead of forward-simulating fragile open-loop controls. Indeed it was shown by Thijssen and Kappen (2015) that the optimal importance sampler coincides with the optimal feedback controller. Therefore, if our ancillary controller is already close to the optimal policy, the sampling procedure yields mostly informative samples and the estimated control command will be close to the optimum.

If no such policy is available *a priori*, we can use our same algorithm to train a parametrized policy with machine learning: based on the cross-entropy formulation of PI control, Kappen and Ruiz (2016) derive an update rule for the general class of sampling policies $\pi_{\theta }(t,x)$ that are parametrized by the vector θ . An alternative derivation of a sum-of-squares error function by Farshidian and Buchli (2013) yields the same update equation. The overall idea is to update the sampler’s parameters at each iteration of the PISOC algorithm with information from the currently sampled trajectories. The update rule is similar to the behavior cloning setup of IL

(30) $$\theta^{+}=\theta +\alpha \nabla_{\theta }\sum_{\mathrm{samples}\;i}\int_{t_{0}}^{t_{f}}\frac{1}{2}w_{i}∥{\tilde{u}}_{i}(\tau )-\pi_{\theta }(\tau ,x){∥}^{2}d\tau$$

where α is a learning rate and $\tilde{u}(\tau )$ the effectively applied control command (including noise) in rollout i at time τ .

In this work, our parametrized sampling policy is a feedforward neural network, but one may apply the same principle to other parametrized function classes such as motion primitives. For the case of neural networks, the gradient updates can conveniently be calculated and applied through standard machine learning software frameworks.

Similar to an off-policy policy search algorithm in the RL context, the parametrized policy can learn from any sample irrespective of the underlying controller that was used during the rollout to produce the noisy controls $\tilde{u}$ . In contrast to policy search, however, our learned importance sampler does not need to become a stable feedback controller before it becomes useful in guiding the sampling process towards low-cost regions. The reason is that it is still “deployed” as an importance sampler in a controlled diffusion process (28), thereby merely guiding the samples instead of dictating the final controls directly.

On the practical side, we note that the update rule (30) can lead to overfitting to a small part of the state space when the system remains stationary for an extended time period. This effect is not unsurprising, considering that the distribution training data becomes extremely centered around the current state. Unfortunately, such uniform data may result in catastrophic forgetting of how to behave in other parts of the state space. Inspired by prioritized experience replay methods in RL (Lin, 1992; Schaul et al., 2016), our countermeasure is to keep a history of the best performing sample for each of the last 500 time steps in memory and include them in the policy gradient computation.

## 5. Implementation

**Figure 1.**
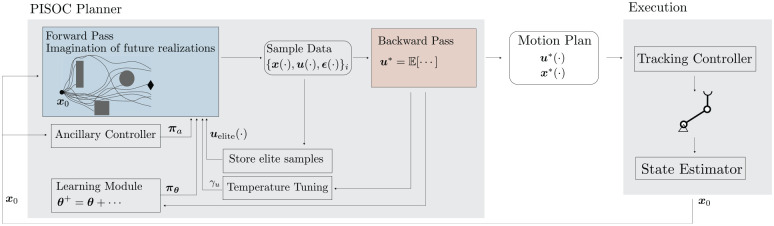
Schematic overview of the components and data flow of our proposed algorithm. The PISOC planner consists of two main routines, namely the forward and backward pass. Its result is a motion plan that can be tracked on a physical robot.

For the application of our PISOC algorithm on real robotic systems, we devise a software implementation that is agnostic to the problem-specific components such as the dynamics, cost function, and constraints. A schematic overview of the components of the method is depicted in Figure 1. The algorithm is included as a solver module in the *Optimal Control for Switched Systems (OCS2)* toolbox.^2^ The key steps of the algorithm at each control cycle are outlined in Algorithm 1.

**Table:** 

**Algorithm 1** Single iteration of our PISOC algorithm
**1: Given:** Current time $t_{0}$ , current state $x_{0}$ **2: Given:** Ancillary or parametrized policy π **3: Initialize:** $\gamma_{u}=\gamma_{J}$ , $U_{\mathrm{elite}}=\emptyset$ **4: Forward Pass:** 5: clear sampleData 6: Sample stochastic trajectories around π 7: Sample stochastic trajectories around $u_{\mathrm{elite}}$ 8: Save all samples to sampleData **9: Backward Pass:** 10: Compute path cost S for all samples 11: Evaluate opt. control $u^{*}(\tau )=E[\cdots ]\;\forall \tau \in [t_{0},t_{f}]$ 12: Roll out $u^{*}(\cdot )$ to obtain $x^{*}(\cdot )$ 13: Save elite samples $U_{\mathrm{elite}}=\{u_{j}(\cdot ){\}}_{j}$ 14: $\gamma_{u}\leftarrow$ temperature tuning **15: if** π parametrized **then** 16: Apply gradient update $\theta^{+}=\theta +\cdots$ 17: **end if** 18: **return** Optimal input and state sequence $\left\{u^{*}(\cdot ),x^{*}(\cdot )\right\}$

### 5.1. Forward–backward pass

The computational procedure can be decomposed into two distinct phases, similar to OC shooting methods. First, our forward pass generates samples by rolling out the controlled stochastic dynamics. Sampling these trajectories can be thought of as a process of imagining possible future realizations. A discrete-time version of (28), which is suitable for implementation on a digital computer, reads

(31) $$x[k+1]=x[k]+(f+G\pi_{a})\Delta t+\mathrm{GQ}\Delta w$$

where we have used the Euler–-Maruyama discretization method with a fixed time step Δt for simplicity. All state-dependent quantities are evaluated at x[k] and the noise increment is sampled according to Δw~N(0,ΔtI) . The covariance matrix Q is determined from (19) according to the currently active constraints and control costs.

During each rollout, we record a trajectory of states, inputs, noise increments, and stage cost. Should the system’s state diverge during the simulation, we directly stop the time integration and remove this sample from the memory because its contribution to the expectation would have been negligible in any case. The entire forward-pass procedure is implemented as a set of independent tasks (one per sample), which can be processed in parallel by several worker threads.

Afterwards, the backward pass is processing the batch of samples. The stage cost of each sample is accumulated from the final to initial time, providing a cost-to-go S for each time step. The relative importance of each sample and the resulting OC can subsequently be computed according to (23) and (24) for all time steps. Finally, the optimized control inputs and reference trajectory are transmitted to the robot to be tracked until the next iteration of PISOC is complete. Note that, despite suggested by Williams et al. (2018a), we do not require any control signal smoothing and no such filter is implemented in our experiments. We suspect that the auto-tuning mechanism of the temperature parameter $\gamma_{u}$ ensures that the averaging effect of many samples is always large enough to forgo additional smoothing.

### 5.2. Importance sampling

All but the simplest systems under our consideration require a form of importance sampling to achieve the required sampling efficiency for real-time control. Apart from the mentioned options of simply re-using the optimal input sequence from the previous iteration, we experiment in Section 7 with using a deterministic sequential linear-quadratic (SLQ) MPC solver based on Farshidian et al. (2017) and Grandia et al. (2019) as ancillary controller. To avoid additional delays in our MPC iteration, we calculate such a computationally intense sampling policy *in parallel* to the forward pass as yet another independent task. The employed sampling policy is, therefore, always the one computed during the previous iteration of our algorithm.

Furthermore, in experiments relating to a learned policy for importance sampling, we use a standard, fully connected feed-forward neural network architecture independent of the system at hand. The network has a single hidden layer with tanh activation and twice as many neurons as inputs.

## 6. Illustrative example

**Figure 2.**
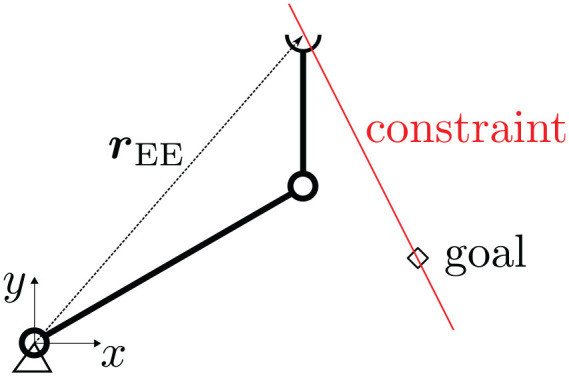
The planar, 2D robot arm considered as an illustrative example. The red constraint line indicates that the end-effector must remain on the direct connection between start and goal position.

We use a simple, illustrative example to visually present some core properties of our algorithm. Consider a two-link, fixed base robot arm as depicted in Figure 2. We want to model and control this system kinematically. Therefore, the state $x\in R^{2}$ of the robot contains the two joint angles and the control input $u\in R^{2}$ corresponds to the commanded joint velocities. The cost function penalizes control effort and encourages the end-effector to arrive at the goal location at the final time. We choose a time horizon of 1 second and an integration time step of 10 ms. In summary, the overall SOC problem reads

(32) $$\begin{array}{l} \underset{u(t_{0}\ldots t_{f})}{\mathrm{minimize}}\;E_{x_{0}}\{\int_{t_{0}}^{t_{f}}∥u{∥}_{R}^{2}d\tau \\ +10^{3}∥r_{\mathrm{EE}}(t_{f})-r_{\mathrm{goal}}{∥}^{2}\}, \end{array}$$

(33) $$\mathrm{subject}\;\mathrm{to}\;x(t_{0})=x_{0}$$

(34) dx=udt+Q(t,x)dw

where Q is defined according to (19) with noise level $\gamma_{J}=0.1$ . The control cost weight models a situation where the first joint is more costly to move: R=diag(10,1) . Given this cost function, we intuitively expect the optimal solution to almost exclusively use the elbow joint only, because its associated control cost is much smaller than that of the shoulder joint.

**Figure 3.**
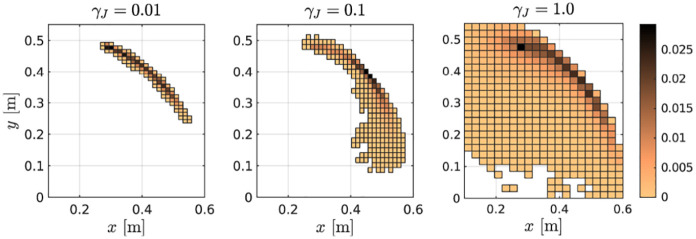
Comparison of sample exploration across different noise levels. The plots show a 2D histogram of task-space positions encountered by the samples. The shading corresponds to empirical probabilities.

We begin by investigating the effects of different noise levels $\gamma_{J}$ on this unconstrained problem. In Figure 3, one can observe the relative frequency of task-space positions that are encountered by the sampling process for the unconstrained system without any importance sampling scheme. For very low noise levels, the shoulder joint is hardly excited by the perturbations and, subsequently, the bulk of the samples are concentrated around a circular arc around the elbow joint. As the noise level increases, more and more samples begin exploring a larger proportion of the state-space and, therefore, have a better chance of discovering possible solutions to the problem. For the same noise levels, Table 1 lists the effective proportion of samples that contribute to the solution. For the nave $\gamma_{u}=\gamma_{J}$ choice, the effective sample size correlates positively with the noise magnitude because the 10-fold increase in the noise dominates any variations in the path costs. Counterintuitively, despite samples spreading further into sub-optimal regions (see Figure 3: more samples explore the region close to the origin, which is not expedient here), a larger proportion of samples contributes to the optimal solution. We can avoid this unfortunate dependence on the absolute noise magnitude with our auto-tuning mechanism. Subsequently, our numbers show an opposing trend: as samples spread further, a smaller fraction of samples ends up in low-cost regions. Therefore fewer of them contribute to the overall solution. At the same time, we are automatically keeping the relative sample size at a level where still sufficiently many noise realizations are averaged to produce a smooth control command.

**Table 1.** Comparison of effective sample size (in per cent) for different noise levels and different choices for the $\gamma_{u}$ parameter

$n_{\mathrm{eff}}$	$\gamma_{J}=0.01$	$\gamma_{J}=0.1$	$\gamma_{J}=1.0$
$\gamma_{u}=\gamma_{J}$	0.10%	0.24%	2.73%
Auto-tuned	37.9%	33.8%	29.1%

We now introduce an additional constraint on the end-effector motion by requiring that the end-effector shall only move along a straight line between current position and goal (shown in red in Figure 2). Such a specification can be motivated through safety concerns, e.g., because the permissible workspace of the robot is limited or because it is preferred that the robot moves in a predictable way. The scalar constraint equation is

(35) $$d_{c}^{⊤}\left[\begin{array}{cc} 0 & 1\\ -1 & 0 \end{array}\right]J_{\mathrm{EE}}\;u=0$$

with $d_{c}:=r_{\mathrm{goal}}-r_{\mathrm{EE}}$ describing the direction along the constraint and $J_{\mathrm{EE}}$ being the end-effector Jacobian that fulfills ${\overset{\cdot }{r}}_{\mathrm{EE}}=J_{\mathrm{EE}}\overset{\cdot }{x}$ .

**Figure 4.**
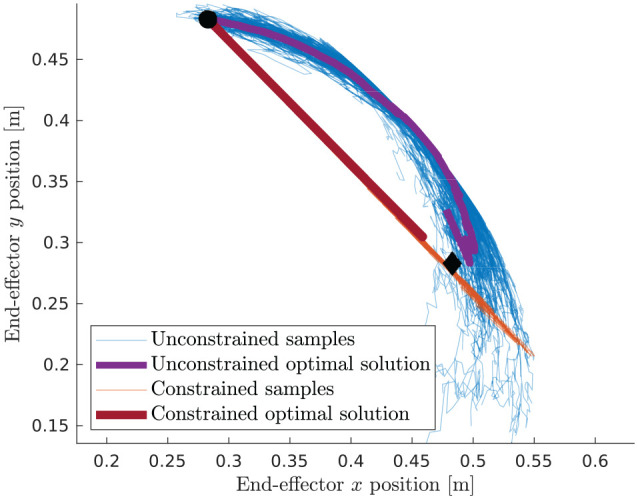
Visualization of sampled trajectories and the computed optimal solutions to the illustrative problem for the constrained and unconstrained case. The start and goal locations are marked by a filled bullet (•) and diamond (♢), respectively.

Figure 4 shows sampled trajectories together with the chosen optimal path for the constrained and unconstrained problem (32). It is evident that they both reach the specified goal position, yet the paths they take are very different. Without constraint, as anticipated, the preferred solution uses mostly the cheaper and more noisy elbow joint. When subjected to the constraint, the samples have no other option than using both joints simultaneously to remain on the constraint manifold.

Note that it is unrealistic to expect that the samples exactly end up at the goal location because the initial state is relatively far away from the goal and it takes most samples at least 70 time steps to make their way to the goal. Although increasing the number of samples would help, it is also not a problem in practice because the algorithm usually runs in a receding horizon fashion, hence only the first few time steps are applied to the system before replanning.

**Figure 5.**
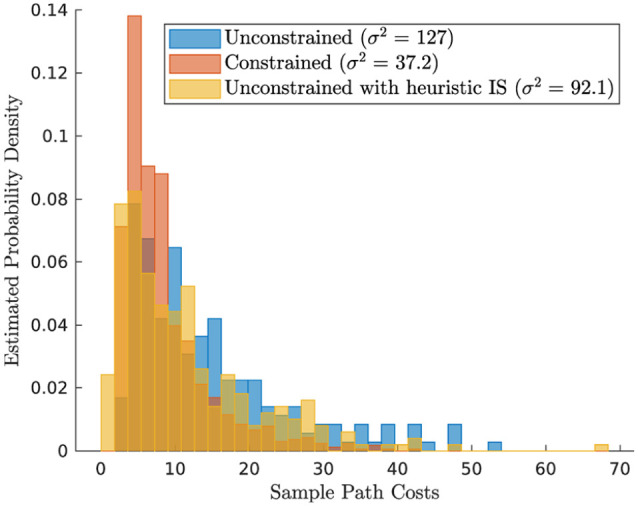
Distribution of sample path costs for the illustrative problem in different scenarios: unconstrained, constrained, and unconstrained with heuristic importance sampling policy.

Turning to the path cost distribution of the samples, Figure 5 reveals that the best score achieved by any sample of the constrained problem is worse than the best score achieved in the unconstrained setting. This is expected because the constraint forces the system into a sub-optimal path with unnecessarily expensive shoulder joint motion. Important to note, however, is that the variance of the underlying sampling procedure is reduced by a factor of 3.4 in the constrained scenario. This variance reduction effect will gain more significance as more complicated (unstable, higher-dimensional) systems are considered in the following sections: While the optimal solution for the unconstrained 2D system in this section can still be found with a manageable number of samples, the sampling space in higher dimensions quickly grows beyond computational limits. We, therefore, regard it as vital to leverage constraints in order to reduce the sampling complexity and to generate more reliable, low-variance solutions at the expense of injecting some bias.

As a final showcase on this illustrative problem setting, we demonstrate that the introduction of a very simple importance sampling heuristic can already have noticeable effects on the sampling performance. To support this point, we introduce the importance sampling policy $\pi_{a}=\left[0,\;u_{2}\right]$ , which solely moves the elbow joint at a fixed rate $u_{2}$ such that it would end up in a configuration close to the desired one in the absence of noise. Figure 5 shows that this simple heuristic already has visible effects on the sample distribution of the unconstrained system, which is now peaked at a better cost value. This suggests that the importance sampling policy need not be an elaborate controller to already have a significant effect on the sampling distribution.

## 7. Results

In this section, we apply the algorithm that is presented in Sections 4 and 5 to two unstable robotic systems. Extending the preliminary outcomes of the illustrative example, the following simulation and hardware results showcase the applicability of the method to a broad spectrum of robot designs and environments.

The noise level $\gamma_{J}$ for each system is kept constant across all experiments unless specifically mentioned. Our rule of thumb to find an appropriate value for this scalar parameter is to increase it as much as possible for maximum exploration while keeping it limited to a level where the system remains controllable. Furthermore, the planning horizon $t_{0}-t_{f}$ is 1 second for both problems.

In both of the following examples, the internal dynamics (1) are implicitly given by the evaluation of a physics simulator. Specifically, we use the physics engine RaiSim (Hwangbo et al., 2018) to forward-simulate the dynamics. This simulator was shown to perform fast and accurately with articulated robotic systems. In addition, it is simple to model the environment in the form of a heightmap inside the simulator in order to evaluate the performance of our algorithm in more realistic scenarios. In experiments that involve a heightmap, this map is predefined (either randomly generated or manually modeled) and static because a continuous perception and mapping pipeline is out of the scope of this work.

### 7.1. Ballbot: a ball-balancing robot

**Figure 6.**
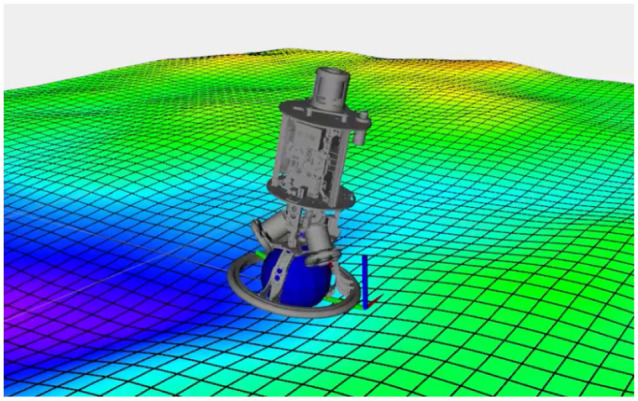
The Ballbot robot operating on randomly generated sloped terrain. The target position (origin) is marked by the coordinate system.

In this section, we show simulation results for Ballbot (Figure 6), a ball-balancing robot . Its state comprises 10 DOF, corresponding to the planar position and 3D orientation of the base, as well as the time derivatives of said quantities. The system has three control inputs that match the torques applied between platform and ball. These commands can directly be passed to the simulation for forward integration without needing an intermediate tracking controller.

The OC problem that we are considering for this system has no constraints and a cost function of the form

(36) $$\begin{array}{c} S(t_{0},x(\cdot ),u(\cdot ))=∥x(t_{f})-x_{\mathrm{ref},f}{∥}_{H_{f}}^{2}\\ \;+\int_{t_{0}}^{t_{f}}\frac{1}{2}(∥u(\tau ){∥}_{R}^{2}+∥x(\tau )-x_{\mathrm{ref}}(\tau ){∥}_{H}^{2})d\tau \end{array}$$

where the time-dependent state reference $x_{\mathrm{ref}}$ is fixed to a nominal stationary state except for the linear base position and velocity components, which are determined by the operator’s command. Finally, the low-level control loop on the robot is merely tracking the commanded torques on the ball as commanded by the PISOC optimizer, absent of any state feedback or pre-stabilization.

In the following, we consider different importance sampling policies for the Ballbot SOC problem. One of them is an existing SLQ MPC algorithm developed by Minniti et al. (2019), which we denote with $\pi_{a}$ (actively controlled sampling policy). This algorithm internally uses a system model based on the rigid-body dynamics equations of motion. Importantly, the deterministic OC is computed under the assumption of perfectly flat ground, no slippage between the ball and the ground, and no actuator effects (Farshidian et al., 2014) . Although the resulting ancillary policy should be a good sampling prior (Thijssen and Kappen, 2015), the resulting estimator is generally not optimal owing to the restrictive modeling assumptions and the fact that no stochasticity is considered. We can expect, however, that the generated samples are in the vicinity of an optimal solution and, therefore, serve as a meaningful comparison for other sampling strategies in the following experiments.

#### 7.1.1. PISOC performance

Despite the instability of the uncontrolled system, our algorithm manages to control the Ballbot even without an externally provided sampling policy. The relative ease of stabilization can be attributed to the fact that the system has an equilibrium point at the default (upright) state with zero input. Therefore, even the default passive $\pi_{p}$ policy automatically explores in all directions and yields sufficiently informative samples to compute an optimal stabilizing policy.

Although not strictly necessary, it is nonetheless insightful to observe the behavior of our algorithm when adding a neural network as parameterized importance sampling policy $\pi_{\theta }$ . In the case of Ballbot on flat ground, we observe that approximately 500 gradient update steps (i.e., controller time steps) are sufficient to make the policy converge to a stable importance sampler. Surprisingly, once converged, the sample size can be even be reduced to one, meaning that the learned sampler has converged to a stable feedback controller and is, therefore, a zero-variance estimator of the optimal input (22).

In order to make the task more interesting, we place the robot on hilly terrain, as shown in Figure 6. Under this condition, we observe that the system remains stable but does not converge to its target when standing on sloped ground. Remarkably, even when employing the SLQ ancillary controller $\pi_{a}$ a noticeable offset persists. The reason is that sufficiently steep slopes cause all rollouts that evolve around terrain-unaware policies to drift downhill, thereby creating a biased sampling distribution. In the following, we show that learning an importance sampling policy provides a remedy to this drift problem.

#### 7.1.2. Online learning

The attempt to learn a parametrized importance sampling policy of the form $\pi_{\theta }(x)$ for the Ballbot system on uneven ground would make the policy specific to the exact terrain that is used during training, thereby preventing generalization to unseen environments. Instead, more practical and interesting is the application of parameter updates (30) during online control. Indeed, we find that the learned sampler’s long-term performance in stabilizing the system with a small number of samples is significantly improved if the gradient updates (30) continue to be applied beyond an offline burn-in phase.

The receding-horizon application of our algorithm with learned importance sampling can be interpreted as a form of online learning: in contrast to learning a globally optimal policy before deployment, our sampler continuously adapts to the changing environment and state that it encounters at runtime. In particular, in the case of learning a linear state feedback controller, the update rule (30) can be interpreted as a form of integral controller that accumulates information from the state history. Different to a standard integral controller, however, is that the feedback signal to our learner is the error between estimated OC and applied control instead of the state tracking error. Even when the state error persists, this crucial distinction prevents the learner from experiencing unbounded integrator windup.

**Figure 7.**
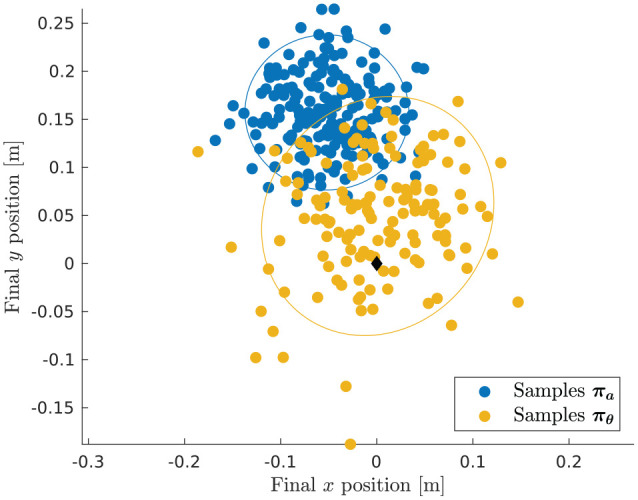
Terminal locations of samples from the SLQ ancillary controller $\pi_{a}$ and the learned sampling policy $\pi_{\theta }$ for the Ballbot example on sloped terrain. The ellipses indicate an uncertainty bound of ± two standard deviations. Only samples that are actively stabilized and that push against the slope end up close to the goal ( ♢ ) and achieve a high reward.

For the Ballbot system, the advantages of the integrating behavior can be seen when encountering inclined terrain. Figure 7 shows that the samples that are unaware of the local terrain end up in locations far away from the goal at the origin. The learned controller, on the other hand, understands that a bias force needs to be applied to fight against the inclination. Therefore, despite being unaware of the terrain directly, the learned policy uses the information of previous rollouts to infer that it is advantageous to apply a bias force against the slope.

Finally, we also observe a failure mode of continuous learning in our experiment: when the system remains at a fixed state for longer periods of time, the collected data may eventually be harmful because the learner experiences local overfitting to the current state. This failure case emphasizes the need for our proposed replay memory structure to promote generalization. In addition, the PISOC structure easily allows switching the sampling policy to a fallback controller, which should be used in case a sudden drop in effective sample size is detected.

### 7.2. ANYmal: a quadrupedal robot

**Figure 8.**
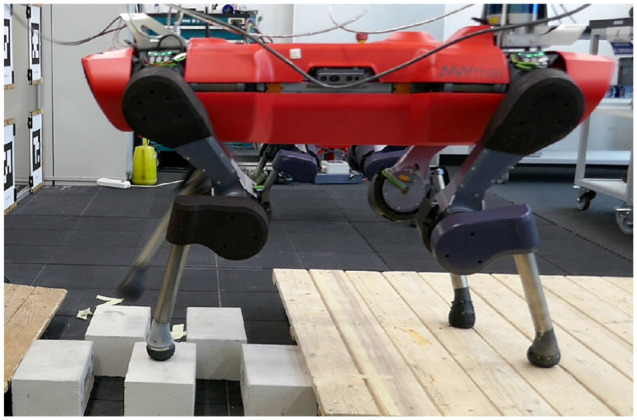
The quadruped ANYmal traversing a stepping stone terrain while being controlled by our proposed algorithm

Our second system is the quadruped robot ANYmal, depicted in Figure 8, for which we present hardware results . With 12 joints, 4 contact points, and a floating base, this system is significantly more complicated and unstable than Ballbot.

For the purpose of forward-simulating stochastic rollouts, we again use the RaiSim physics engine. Internally, the simulator treats ANYmal as an 18-DOF articulated system and expects a 12-dimensional control input, corresponding to the joint torques. However, to make our algorithm comparable to a deterministic MPC benchmark, the control space that the PISOC algorithm operates on is only a kinodynamic one. This means the control input $u\in R^{24}$ contains the contact forces $f_{\mathrm{EE}}$ that act at each foot as well as the joint velocities. A full description of the kinodynamic model can be found in Grandia et al. (2019). A standard inverse dynamics controller computes the transformation from these controls to joint torques. This controller and the simulator together are represented by the dynamic model quantities f,G . A subtle but important feature of this formulation is that the state of the system as seen by the PISOC algorithm evolves continuously because the joint velocities, which may be affected by an impact, are not part of the kinodynamic state.

We use a quadratic cost function analogous to (36). The only difference is that the control weight matrix R penalizes velocities in the operational space of the foot instead of joint space. Importantly, this allows shaping of the noise magnitude in the Cartesian directions of the foot motion, which is more intuitive than doing so in joint space.

In this example, constraints are used to encode a pre-defined gait sequence that we want the walking robot to obey: support legs are forced to be stationary in the world reference frame while swing legs must not apply any contact force and are made to follow a predetermined velocity profile s(t) in the direction perpendicular to the ground:

(37) $$v_{\mathrm{EE},i}=0\;\;\;\;\;\;\;\mathrm{for}\;i\in \mathrm{stancelegs}$$

(38) $$v_{\mathrm{EE},i}\cdot \hat{n}=s(t),\;f_{\mathrm{EE},i}=0\;\mathrm{for}\;i\in \mathrm{swinglegs}$$

Owing to the kinodynamic input vector, these conditions can be formulated in the control-affine form (12). For all following investigations on ANYmal, the switching times between stance and swing legs are assumed to be given according to a user-defined gait pattern.

For ANYmal there also exits a SLQ MPC implementation by Grandia et al. (2019), which we use as a baseline active sampling policy. For this deterministic OC solver the robot is modeled as a kinodynamic system with 24 states and 24 inputs. Kinodynamic in this context means that the torso of the robot is approximated as a single rigid body to which massless legs are attached. Thus, the state includes the pose and twist of the robot’s base as well as three joint positions per leg. The control input corresponds to the foot contact forces and the joint velocities, just as introduced for the PISOC problem above.

The increased complexity of the problem forces us to execute the PISOC algorithm on a separate computer, distinct from that which runs the state estimator and tracking controller (see Figure 1: physical separation of the shaded boxes). With a well-performing importance sampler, we find that drawing 64 samples in each iteration is a good compromise between computation time and solution quality and smoothness. On the robot side, the optimized kinodynamic state and control trajectories are translated into joint torques through a hierarchical inverse dynamics controller (Bellicoso et al., 2017). The tracking controller and PISOC algorithm run asynchronously on different machines and subsequently always operate on the most recent state or OC solution available. This physical separation makes the control task more difficult owing to additional network delays.

#### 7.2.1. Importance sampling is critical

The illustrative problem in Section 6 already showed that even a straightforward importance sampling scheme has the potential to improve the effective sample size. Now, we demonstrate that this effect becomes more pronounced for systems with more DOF.

**Figure 9.**
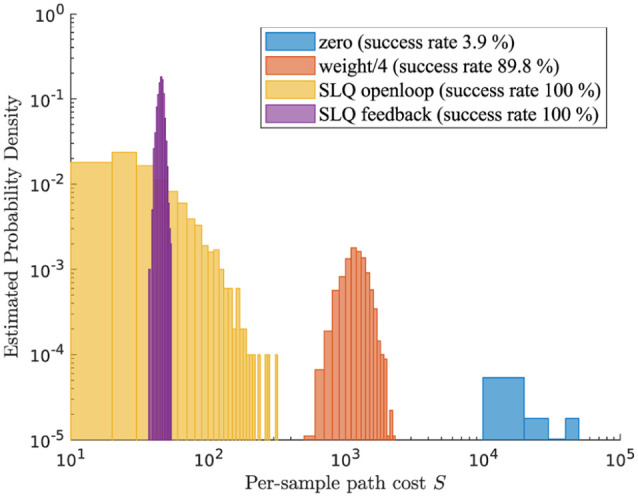
Histogram visualization of the distribution of sampling path costs. We compare different ancillary controllers for the task of stabilizing a standing quadruped.

In Figure 9 we plot the sampling performance under different importance sampling policies $\pi_{a}$ for the task of stabilizing a standing quadruped. We compare four different options for importance sampling: the default constrained policy of sampling around zero contact forces ( $\pi_{c}$ , “zero”), a simple heuristic input that commands zero joint velocities and constant contact forces that compensate the weight of the robot equally (“weight/4”), open-loop SLQ MPC policies (“SLQ openloop”), and feedback SLQ MPC policies (“SLQ feedback”).

Several important insights can be gained from this figure. First, even a simple pre-stabilization such as gravity compensation already improves the average sample performance by an order of magnitude. Second, another order of magnitude improvement can be gained by computing a control sequence that is aware of the current state and system evolution, as SLQ does it. In the presence of noise and a complex, unstable system, it is otherwise extremely unlikely to find good samples. Finally, a sampling policy with feedback drastically reduces the variance of the sampling variance because random perturbations can already be compensated during the rollout. However, the price for feedback is a lack of exploration, which prevents discovering trajectories that may achieve even lower costs.

When repeating the same experiment for a walking motion, we find that it is virtually hopeless to produce a good sample without an appropriate sampling policy for a system that is both unstable and has a large sampling space. The reader is referred to the supplementary video^3^ for a demonstration.

In summary, our results provide empirical evidence that importance sampling is not only helpful but rather vital when the complexity and instability of a system grows. Conveniently, the structure of PISOC allows almost any existing controller to be incorporated as an importance sampler, making importance sampling almost no additional effort for many existing systems.

#### 7.2.2. Learning importance sampling

Having established that using an ancillary controller as sampling policy can significantly improve the sampling efficiency, we now show that such a policy can also be learned with our PISOC algorithm. Doing so is particularly helpful for online control if the ancillary controller is not able to run in a real-time setting. In addition, learning can be useful because the trained policy can be conditioned on additional observations beyond the system’s state. We make use of this possibility when training a sampler for the ANYmal system. The augmented observation that serves as input to the neural network also includes a phase variable and contact flag for each leg that encodes the requested gait. For a fair comparison, the state x that the learned policy receives is the same kinodynamic state that is used by the SLQ optimizer instead of the full dynamic state from the simulator.

**Figure 10.**
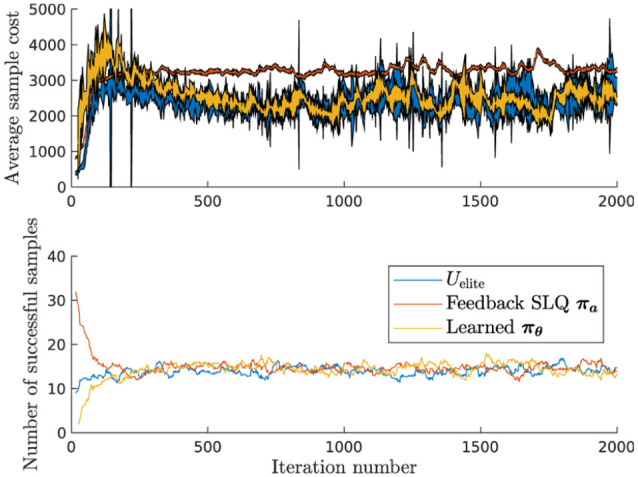
Comparison between different importance sampling strategies for ANYmal under a static walk gait. The learned importance sampler is started with random parameters and is updated once per iteration. The top plot shows the average sampled path cost (lower is better) with the width of the shaded lines corresponding to ± two standard deviations. The bottom plot indicates the number of successful samples.

To assess the quality of the sampling procedure, we compare average sample path cost, standard deviation of the costs, and total number of successful samples. An optimal sampler would consistently draw a sample with the lowest possible cost for the given starting state and noise realization. The progression of learning a sampling policy $\pi_{\theta }(t,x)$ for a static walk motion of ANYmal from randomly initialized parameters can be seen in Figure 10. It can be observed that already after roughly 300 iterations (i.e., 300 gradient updates), the learned sampler begins to outperform the SLQ policy in terms of average reward and is on par with the number of successfully completed rollouts. A successful sample in this context is one that did not diverge numerically and did not end up in an unrecoverable state. The average reward of rollouts around the last iteration’s best input sequences ( $U_{\mathrm{elite}}$ ) returns scores that are similar to the best importance sampling strategy, on average. The SLQ policy is biased towards a solution that is only optimal with its internal deterministic kinodynamic model and not the stochastic, simulated dynamics. However, its aggressive feedback gains yield very consistent sample returns independent of the noise realization, making it an especially low variance sampling policy. The fact that the spread of $\pi_{\theta }$ is smaller than that of the open-loop elite samples indicates that the learned policy also developed some form of state feedback, which allows it to counter noise perturbations during a rollout. Visually, all three policies have very similar behavior.

These promising results can be reproduced for different gaits on ANYmal. However, we also note that the behavior of the learned sampling policy is only valid in the neighborhood of the data that it was presented. For unseen motions, e.g., larger setpoint changes than trained with, the learned sampler cannot consistently outperform the SLQ policy. This is due to the well-known problem of distribution mismatch between training and test data. Fortunately, the sampling procedure of PISOC acts as a form of safety net that does not assume that the importance sampler is an optimal policy in all states and hence provides an extra layer of robustness is these situations.

#### 7.2.3. Constrained problems

**Figure 11.**
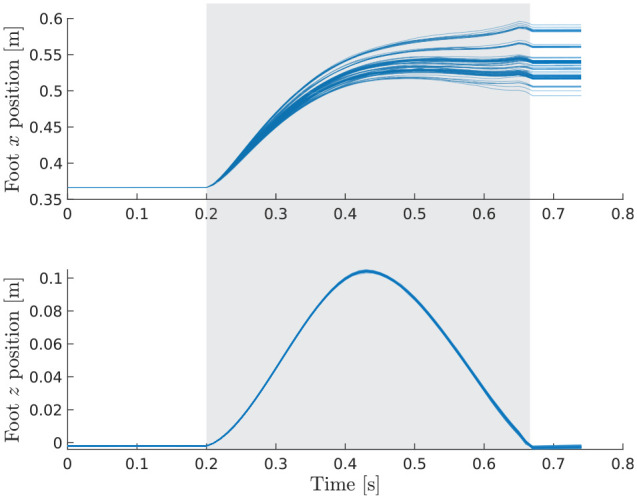
Sampled foot trajectories for one swing phase. The shaded area indicates the time in which the swing constraint is active.

Through our use of projection matrices into the constraint null space for both the controlled input (18) and (27) and the noise (19), it is guaranteed by design that all samples fulfill the constraint (12). The OC is a convex combination of the noisy controls applied during sampling. Therefore, also the OC satisfies the affine constraint. In Figure 11, we validate this statement for the ANYmal example by plotting the task-space trajectories of one foot. From the figure, it is evident that the swing constraint ( z direction) is fulfilled because all sampled trajectories coincide. In the other directions, the samples are free to explore in order to find suitable foothold positions, here shown exemplarily in the x direction. Note also that once a swing phase is complete, the feet are forced to remain stationary, which is individually enforced for each sample.

The illustrative example in Section 6 has already demonstrated that constraints can significantly reduce the sampling complexity and that this variance reduction usually comes at the expense of a biased solution. In the case of ANYmal, the difference between a constrained and unconstrained problem formulation is especially striking: With gait constraints, the samples reasonably explore where to step to keep balance, as can be observed in Figure 11. When temporarily disabling the constraints (37) and (38), we could not observe a single sample that manages to find a suitable walking motion, even after several iterations of the algorithm. Instead, the optimal solution that could be found is a vibrating leg motion that can hardly keep the balance. Moreover, when issuing a forward-motion command in such an unconstrained setting, the robot begins to shift its body in the desired direction but fails to discover a stepping behavior and eventually collapses. The difference between the constrained and unconstrained setting is best appreciated by watching the accompanying video. The fact that not a single sample discovered how to achieve a lower path cost by stepping forward emphasizes that the use of constraints is an essential mechanism to bound the sampling complexity.

#### 7.2.4. Robustness and exploration over local minima

A key motivation for stochastic, sampling-based algorithms is their ability to explore unknown states and thereby gain robustness and overcome local minima. Figure 3 has already foreshadowed that the injection of noise promotes exploration, yet the cost landscape of the 2D robot arm was too simple to benefit from further exploration.

**Figure 12.**
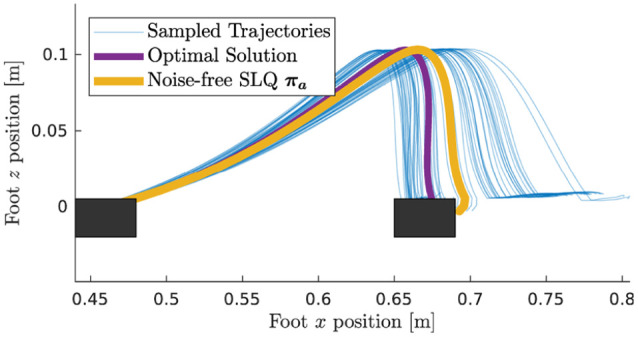
Visualization of sampled foot trajectories in a stepping stone scenario. The dark rectangles represent suitable footholds. The samples on the right-hand side start diverging when no ground reaction force is encountered.

For ANYmal, we now show that there are indeed scenarios where exploration is critical for success. Consider the case where our robot is expected to cross a terrain segment where only a few sparse areas (“stepping stones”) represent suitable footholds. Figure 12 displays the foot trajectories that are proposed by the sampling procedure when a single stone is reachable in the horizon. The visualization shows that the ancillary noise-free SLQ policy (drawn in yellow), which is unaware of the terrain, would have commanded the foot to miss the stepping stone and only hit it on the side. Such samples, however, are quickly rejected because the corresponding state diverges, as can be seen on the right-hand side of the figure. Therefore, despite our importance sampling policy predicting a fatal move, it is through the exploration that we are able to probe the surrounding area and discover safe footholds. In the video, we additionally show the case where the algorithm explores the option to step on different stones and sometimes decides to go zero, one, or two stones forward in a single swing phase. This result shows that sampling-induced exploration is a powerful mechanism for reacting to unforeseen circumstances and refining the underlying importance sampling policy.

Another interesting investigation is the relationship between the noise level and the robustness against unseen environments. In Table 2, we relate the size of the gaps between stepping stones to the noise level that is required to discover the next safe foothold. As the overall magnitude of the noise is dependent on the scale of the cost function, we state the noise magnitude relative to the default value that is used throughout the rest of the experiments on ANYmal. From the table, it can be seen that generally more stochasticity is required as the task becomes harder (that is, further away from the nominal sampling policy). However, there is a limit to how much the noise can be increased because eventually the OC itself exhibits a noise magnitude that is not suitable for tracking anymore and leads to undesired vibration modes on the system.

**Table 2.** Minimum required exploration noise level for crossing gaps of various widths. The largest gap setting requires a noise magnitude that is too high for reliable operation.

Gap width (cm)	15	20	25	30	35
Relative noise level	30%	55%	100%	115%	—

#### 7.2.5. Hardware deployment

Finally, we deploy the proposed algorithm on the physical ANYmal robot. Although the transfer from simulation to reality does not require any additional tricks on the algorithm side, a key challenge is the computational burden of the sampling procedure. The update rate on our machine (AMD Ryzen 9 3950X, 16 × 3.6 GHz) reaches approximately 15 Hz when rolling out 64 samples per iteration. The resulting average policy delay (computation and network transmission) of 0.12 second appears to be just fast enough for real-time control on ANYmal. In the accompanying video, one can observe a robust walking behavior that tracks the user’s commands in terms of displacement and yaw orientation.

Beyond flat-ground walking, we also successfully reproduce the stepping stone task (see Section 7.2.4 and Figure 8) on hardware. The heightmap corresponding to the physical scenario is generated and loaded into the simulator once before execution starts and held static afterwards. For this scenario, too, our video shows that ANYmal reliably walks across the stones. Interestingly, the algorithm even sometimes plans a swing trajectory across two stones when the corresponding path cost is lower. The ease with which complicated scenarios can be modeled inside a physics engine, together with the fact that no algorithmic change is needed, underline the versatility of our approach.

**Figure 13.**
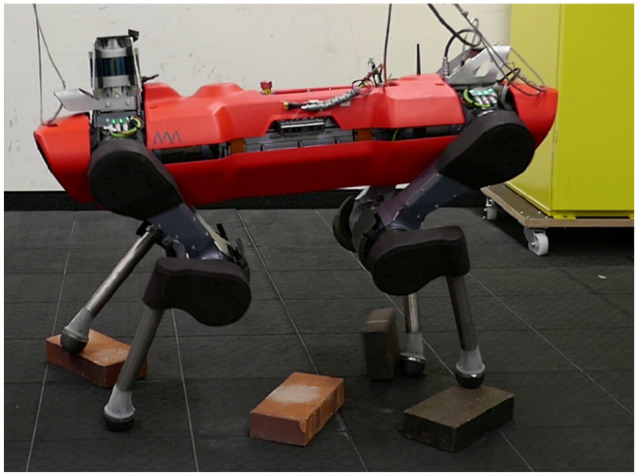
ANYmal system walking blindly across obstacles that are not included in the simulated environment.

Finally, we also verify the robustness against unmodeled ground variations on hardware. To this end, a few stones are randomly placed in front of the robot, as depicted in Figure 13. Upon a forward-walking command from the operator, the robot successfully steps across the obstacles. Interestingly, when the robot steps on an obstacle, thereby violating the planned trajectory, audible vibrations indicate that currently fewer samples contribute to the solution and thereby make the optimized controls noisier. The corresponding scenes can also be seen in the video.

## 8. Conclusion

In this article, we have developed a stochastic control algorithm that is suitable for controlling high-dimensional, non-linear systems. It has been shown that the sampling mechanism waives the need for a differentiable dynamics model, thereby presenting itself with increased flexibility in modeling the system and the environment. In particular, we have demonstrated how a physics simulator can be used to represent the system’s interaction with complex geometries present in the environment.

Further, we have shown that standard Monte Carlo sampling for high-dimensional systems is intractable. Our results provide evidence that the two proposed countermeasures, namely constraints and importance sampling with an ancillary controller, are effective in restoring a workable sampling efficiency.

In addition to reducing the sampling complexity, our extension of PISOC to equality constraints gives the designer an intuitive handle to limit the stochastic exploration to safe and useful behaviors. In terms of future work, we imagine that the extension to path inequality constraints may be another interesting way of incorporating domain-specific knowledge which will boost sampling efficiency. In that regard, we imagine that Lagrange multiplier methods may provide interesting options to work around the restriction of quadratic path costs. Furthermore, it would be interesting to establish a relationship between the degree of simulation–reality mismatch and any performance or safety guarantees of the algorithm.

The mechanism of rolling out stochastic trajectories around an existing ancillary controller also has a secondary benefit: One may re-use existing control schemes and effectively view the sampling procedure as a safety net and refinement mechanism around proved high-performing controllers. For example, one could assign the responsibility for perception and obstacle avoidance to the sampling procedure only, whereas the blind ancillary controller contributes a stable core behavior. When deploying such a setup in practice, care must be taken with regard to the feedback characteristics of the ancillary controller: we observed that using an overly aggressive feedback controller as the underlying sampling policy can have adverse effects on the overall performance because the feedback terms heavily suppress the exploration capabilities of the sampling procedure.

The showcase for learning an importance sampler puts our method yet in another perspective and highlights the versatility of the algorithm: one can imagine use-cases where an importance sampler is first trained in an offline phase, either from scratch (RL) or using an existing controller (IL) and then refined online according to the local sample performance. Our experiments with ANYmal have also shown that a learned policy can be conditioned on further observations, such as the gait. Although the scope of our work was limited to perception-less control, it is not difficult to devise a pipeline where the neural network policy receives additional information from a perception sensor. In that case, the learned sampler could, for example, recognize suitable footholds and steer the samples accordingly. Furthermore, in the online learning scenario, it could be interesting to investigate in the future how the tradeoff between reactiveness and tendency to local overfitting can be balanced automatically.

Our sampling procedure is implemented in parallel on the CPU, making it fast enough for real-time control of a quadrupedal robot, as demonstrated in the previous section. The work of Williams et al. (2017) foreshadows the performance gains that we could realize from also transitioning to a GPU parallelization in the future. Our current limitation in this respect is the availability of physics simulators that can operate on GPU. The recent appearance of GPU-accelerated simulation engines give us reason to expect that a GPU version of our algorithm is realistic in the foreseeable future. Such an implementation would not only make the algorithm faster but also eliminate network delays that arise from having to run the PISOC algorithm on a separate computer.

Lastly, in principle, it is possible to use a sampling-based method to also sample from discrete decisions. This option sounds especially appealing in a robotic context where contacts can be modeled as discrete decisions. For example, this idea may allow us to lift the constraints of a fixed contact schedule and start exploring over the different gaits of a walking robot. Initial experiments in this direction have revealed a fast combinatorial explosion which was not suitable for real-time control, but it may be an exciting research direction to pursue in the future.

## Appendix A. Constrained path integral control derivation

We follow the general PI derivation laid out in Kappen (2005) and extend it to the constrained case.

### A.1. Optimal cost-to-go

The optimal cost-to-go J is the minimal cost (in expectation) that can be achieved when starting from state x at time $t\in [t_{0},t_{f}]$ while respecting the constraint:

(39)
J(t,x)=minu(t…tf)s.t.(12)Ex(t){Φ(xtf)+∫ttf(12u⊤Ru+V(x)+r⊤(x)u)dτ}

To proceed, we split up the time integration into two parts

(40)
J(t,x)=minu(t…tf)s.t.(12)Ex(t){Φ(xtf)+∫tt′(12u⊤Ru+V(x)+r⊤(x)u)dτ+∫t′tf(12u⊤Ru+V(x)+r⊤(x)u)dτ}

The first integration interval only depends on inputs u(t…t′) , so the minimization operation can also be split:

(41)
J(t,x)=minu(t…t′)s.t.(12)Ex(t){×∫tt′(12u⊤Ru+V(x)+r⊤(x)u)dτ+minu(t′…tf)s.t.(12)(12)Ex(t′){Φ(xtf)+∫t′tf(12u⊤Ru+V(x)+r⊤(x)u)dτ}}

Importantly, the second minimization (curly braces) is still inside the first minimization (square brackets) because the state x(t′) depends on the inputs up to t′ . By definition of the cost-to-go (5), one obtains a recursive formula (principle of optimality)

(42)
J(t,x(t))=minu(t..t′)s.t.(12)Ex(t){J(t′,x(t′))+∫tt′(12u⊤Ru+V(x)+r⊤(x)u)dτ}

To transform this recursive equation into a differential equation, we start looking at infinitesimal time intervals and set t′:=t+dt . This allows us to expand the cost-to-go to first order in dt and second order in dx (following Itô calculus rules). We use the short form J:=J(t,x(t)) . For the first term of the above equation, the expansion reads

(43)
$$\begin{array}{c} J(t+dt,x(t+dt))\approx J+(\partial_{t}J)dt\\ +(\partial_{x}J)dx+\frac{1}{2}dx^{⊤}(\partial_{\mathrm{xx}}J)dx\\ =J+(\partial_{t}J)dt+(\partial_{x}J)dx+\frac{1}{2}\mathrm{Tr}((\partial_{\mathrm{xx}}J)dxdx^{⊤}) \end{array}$$

For the following steps, note that we can expand dx by making use of the system dynamics (1):

(44)
$$\begin{array}{c} dxdx^{⊤}=(f+\mathrm{Gu})(f+\mathrm{Gu}{)}^{⊤}dt^{2}\\ +(f+\mathrm{Gu})dtdw^{⊤}(\mathrm{GQ}{)}^{⊤}\\ +\mathrm{GQ}dw(f+\mathrm{Gu}{)}^{⊤}dt\\ +\mathrm{GQ}dwdw^{⊤}(\mathrm{GQ}{)}^{⊤} \end{array}$$

We now substitute this expansion into the expectation while ignoring any terms above first order of dt , yielding

(45)
$$\begin{array}{c} E_{x(t)}\{J(t+dt,x(t+dt))\}\\ \;=E_{x(t)}\{J+(\partial_{t}J)dt+(\partial_{x}J)(f+\mathrm{Gu})dt\\ \;+(\partial_{x}J)\mathrm{GQ}dw\\ \;+\frac{1}{2}\mathrm{Tr}\left((\partial_{\mathrm{xx}}J)\mathrm{GQ}dwdw^{⊤}{\left(\mathrm{GQ}\right)}^{⊤}\right)\} \end{array}$$

Finally, we can compute the expectation using Itô rules and Brownian motion properties $E[dwdw^{⊤}]=Idt$ and E[dw]=0 :

(46)
$$\begin{array}{c} E_{x(t)}\{J(t+dt,x(t+dt))\}\\ \;=J+(\partial_{t}J)dt+(\partial_{x}J)(f+\mathrm{Gu})dt\\ \;+\frac{1}{2}\mathrm{Tr}((\partial_{\mathrm{xx}}J)\mathrm{GQ}Q^{⊤}G^{⊤})dt \end{array}$$

Regarding the second term in the minimization of (42), the integral disappears in the limit dt→0 and the expectation can be dropped because x(t) is the known starting state. Therefore, the recursive formula (42) turns into the deterministic optimization problem

(47)
J=minu(t)s.t.(12)((12u⊤Ru+V(x)+r⊤(x)u)dt+J+(∂tJ)dt+(∂xJ)(f+Gu)dt+12Tr((∂xxJ)GQQ⊤G⊤)dt)

Finally, divide by dt , cancel J , and move terms out of the minimization that do not depend on u to arrive at

(48)
−(∂tJ)=minu(t)s.t.(12)(12u⊤Ru+r⊤(x)u+(∂xJ)(Gu))+V(x)+(∂xJ)f+12Tr((∂xxJ)GQQ⊤G⊤)

### A.2. Minimization under constraints

In this section, we minimize the right-hand side of the above equation using the method of Lagrange multipliers to account for the constraint. Define the Lagrangian

(49)
$$\begin{array}{c} L=\frac{1}{2}u^{⊤}\mathrm{Ru}+r^{⊤}(x)u+(\partial_{x}J)(\mathrm{Gu})\\ +\nu^{⊤}(c(x)+D(x)u) \end{array}$$

where ν is the vector of Lagrange multipliers for constraint (12). The stationarity conditions with respect to u and ν read

(50)
$$\mathrm{Ru}+r(x)+G^{⊤}(\partial_{x}J{)}^{⊤}+D^{⊤}\nu =0$$

(51)
c+Du=0

The matrix R is positive definite, so we can solve the first equation for u and insert it into the second:

(52)
$$c-DR^{-1}\left(r(x)+G^{⊤}{\left(\partial_{x}J\right)}^{⊤}+D^{⊤}\nu \right)=0$$

Under the assumption of full row-rank of D we can solve for ν and plug it back into u :

(53)
$$\nu ={\left(DR^{-1}D^{⊤}\right)}^{-1}\left(c-DR^{-1}(r(x)+G^{⊤}{\left(\partial_{x}J\right)}^{⊤})\right)$$

(54)
$$u=\pi_{c}(x)-(I-\tilde{D})R^{-1}G^{⊤}(\partial_{x}J{)}^{⊤}$$

where we have defined $\pi_{c}(x)$ according to (18). Finally, we plug this result back into (48), perform a number of algebraic simplifications and eventually obtain the HJB equation (15).

### A.3. Transformation and diffusion process

To linearize the HJB equation (15) we apply a logarithmic transformation for the optimal cost-to-go, thereby defining the desirability function ψ :

(55)
J(t,x)=−γlogψ(t,x)

(56)
$$\partial_{x}J=-\gamma \frac{1}{\psi }\partial_{x}\psi$$

(57)
$$\partial_{\mathrm{xx}}J=-\gamma \left(\frac{-1}{\psi^{2}}{\left(\partial_{x}\psi \right)}^{⊤}(\partial_{x}\psi )+\frac{1}{\psi }\partial_{\mathrm{xx}}\psi \right)$$

(58)
$$\partial_{t}J=-\gamma \frac{1}{\psi }(\partial_{t}\psi )$$

This variable substitution turns (15) into

(59)
$$\begin{array}{c} \gamma \frac{1}{\psi }(\partial_{t}\psi )=\frac{1}{2}\pi_{c}^{⊤}R\pi_{c}+r^{⊤}\pi_{c}+V(x)\\ -\frac{\gamma }{\psi }(\partial_{x}\psi )(f+G\pi_{c})\\ -\frac{1}{2}\frac{\gamma^{2}}{\psi^{2}}(\partial_{x}\psi )G(I-\tilde{D})R^{-1}G^{⊤}(\partial_{x}\psi {)}^{⊤}\\ +\frac{1}{2}\mathrm{Tr}[-\gamma (\frac{-1}{\psi^{2}}(\partial_{x}\psi )(\partial_{x}\psi {)}^{⊤}+\frac{1}{\psi }\partial_{\mathrm{xx}}\psi )\\ \cdot \mathrm{GQ}Q^{⊤}G^{⊤}] \end{array}$$

If we can find a γ such that

(60)
$$-\gamma G(I-\tilde{D})R^{-1}G^{⊤}+\mathrm{GQ}Q^{⊤}G^{⊤}=0$$

then the terms quadratic in $\partial_{x}\psi$ disappear and the HJB equation becomes a linear PDE. A sufficient condition for this to happen is when the noise is inversely proportional to the control cost and zero in the direction of the constraint, i.e., Equation (19).

The linearized and log-transformed HJB equation then reads

(61)
$$\begin{array}{c} (\partial_{t}\psi )=-(\partial_{x}\psi )(f+G\pi_{c})\\ +\frac{\psi }{\gamma }\left(\frac{1}{2}\pi_{c}^{⊤}R\pi_{c}+r^{⊤}\pi_{c}+V(x)\right)\\ +\frac{1}{2}\mathrm{Tr}\left[-(\partial_{\mathrm{xx}}\psi )\mathrm{GQ}Q^{⊤}G^{⊤}\right] \end{array}$$

According to the Feynman–Kac formula, the solution of this PDE can be obtained by forward simulating a stochastic process

(62)
$$\begin{array}{c} \psi (t,x)=E_{x_{0}}\{\exp(-\frac{1}{\gamma }(\Phi (x_{t_{f}})\\ +\int_{t}^{t_{f}}\left(\frac{1}{2}\pi_{c}^{⊤}R\pi_{c}+r^{⊤}\pi_{c}+V(x)\right)d\tau ))\} \end{array}$$

where the expectation is over all trajectories governed by the diffusion (21).

Finally, the log transformation can now be inverted to obtain a PI formula for the optimal cost-to-go

(63)
$$\begin{array}{c} J(t,x)=-\gamma \log E_{x_{0}}\exp\{(-\frac{1}{\gamma }(\Phi (x_{t_{f}})\\ +\int_{t}^{t_{f}}\left(\frac{1}{2}\pi_{c}^{⊤}R\pi_{c}+r^{⊤}\pi_{c}+V(x)\right)d\tau ))\} \end{array}$$

Inserting this equation into (54) one eventually obtains a PI equation for the optimal input (22).

## Notes

1. Here $\pi_{c}$ is the policy that minimizes the control cost and that naturally drops out of the derivation.

2. See https://leggedrobotics.github.io/ocs2/

3. See https://youtu.be/C7nqZDE5L2o
